# Annual changes in the microeukaryotic community in intertidal sediments, Qingdao, China

**DOI:** 10.1128/spectrum.00467-25

**Published:** 2025-10-31

**Authors:** Yan Ji, Hongbing Shao, Andrew McMinn, Min Wang

**Affiliations:** 1 College of Marine Life Sciences, Institute of Evolution and Marine Biodiversity, MOE Key Laboratory of Evolution and Marine Biodiversity, Frontiers Science Center for Deep Ocean Multispheres and Earth System, Center for Ocean Carbon Neutrality, Ocean University of China 506915 , Qingdao, China; 2 UMT-OUC Joint Centre for Marine Studies, Qingdao, China; 3 Institute for Marine and Antarctic Studies, University of Tasmania 445071 , Hobart, Tasmania, Australia; 4 Haide College, Ocean University of China 12591, Qingdao, China; 5 The Affiliated Hospital of Qingdao University 235960, Qingdao, China; CEA-Genoscope, Evry, France

**Keywords:** microeukaryotic communities, intertidal sediments (muddy and sandy), OTU composition, seasonal variation, trophic functional groups, Qingdao coastal

## Abstract

Microeukaryotic communities in intertidal sediments play an important role in maintaining ecosystem health and resilience, as they are involved in ecosystem services such as nutrient cycling, organic matter breakdown, and the support of diverse marine food webs. However, the seasonal variations in these communities and the differences between communities in muddy and sandy sediments are poorly understood. To address these gaps, surface sediments from both muddy and sandy sediments were collected monthly over a year (4 seasons, 10 months). Microeukaryotic community structure was analyzed using 18S rRNA sequencing, focusing on alpha-diversity, operational taxonomic unit composition, community composition, network structure, and trophic functional group structure. Results show that there were significant monthly changes in community composition, which were closely linked to changes in environmental factors such as temperature, dissolved oxygen (DO), and salinity. Species co-occurrence network analysis showed that interaction numbers and network density peaked in summer and declined in autumn and winter. This study also highlights the different characteristics of eukaryotic communities of muddy versus sandy sediments. Mantel tests show that temperature, DO, and chlorophyll *a* (chl *a*) have a significant influence on eukaryotic communities in muddy environments, while DO, chl *a*, and particulate organic carbon are important in sandy environments. It was found that autotrophs, heterotrophs, and mixotrophs react differently to seasonal climate shifts and different sediment types, with notable peaks in relative abundance for autotrophs in June, heterotrophs in December, and mixotrophs from September to November, as inferred from 18S rRNA amplicon sequencing data. These results will provide an important baseline on the microeukaryotic communities in intertidal sediments, essential for forecasting changes in community structure and ecosystem functions resulting from environmental changes.

**IMPORTANCE:**

This study is the first to report on dynamic changes in microeukaryotic operational taxonomic unit (OTU) composition in intertidal sediments, focusing on differences between muddy and sandy sediments over 10 months and across two different stations. A total of 4,452 OTUs were identified. Our findings offer new insights into how these communities are influenced by environmental factors, seasons, stations (Licun and Zhanqiao), and sediment types (sandy and muddy). Our results show significant monthly changes in community composition, closely linked to environmental factors such as temperature, dissolved oxygen, and salinity. The species co-occurrence network analysis revealed that interaction numbers and network density peaked in summer and declined in autumn and winter.

## INTRODUCTION

Intertidal ecosystems, particularly those associated with soft substrates, play a vital role in maintaining marine biodiversity and supporting ecological functioning (1). These systems experience regular and often extreme changes due to daily tidal fluctuations, making them highly dynamic environments (2). The importance of soft substrate intertidal ecosystems can be broadly categorized into ecological, biological, and socio-economic aspects. Ecologically, these zones act as buffers, protecting coastlines from erosion and storm surges, a function increasingly important in the context of climate change-induced sea-level rise and intensified storm events (3). In addition, they can contribute to water purification, nutrient cycling, and carbon sequestration (4, 5). Biologically, soft substrate intertidal zones are hotspots of biodiversity. They provide critical habitat, breeding grounds, and nurseries for a range of commercially important fish and shellfish (5). Also, some bacteria produce and release chemical cues to attract the settlement of macroalgal propagules and invertebrate larvae (6). Socio-economically, these ecosystems offer significant services, including food provision, recreation, and education (7). They support fisheries and aquaculture, which are crucial for the livelihoods of coastal communities. Furthermore, their esthetic and educational values promote tourism and foster a connection between people and the marine environment (8).

Intertidal ecosystems are dynamic and complex environments that host a diverse array of benthic eukaryotes, including protists (9). Protists, a group of single-celled microbial eukaryotes that includes microalgae, play a pivotal role in maintaining ecological balance and supporting life in these transitional habitats (10). Unicellular algae, such as diatoms and dinoflagellates, are key components of most intertidal protist communities. They are critical primary producers in coastal benthic ecosystems, contributing up to 70% of total primary production and forming the dietary basis for farmed mussels, oysters, and cockles (7, 11). Diatoms in intertidal sediments secrete extracellular polymeric substances, which contribute to biofilm formation that enhances sediment stability (12). In addition to primary production, protists can also act as predators or parasites, serving to establish key associations in the intertidal food web (13, 14). Predatory protists, such as certain ciliates and cercozoans, help regulate the populations of bacteria, algae, and other protists (15). This control is crucial for maintaining balance in microbial communities in sediments, which in turn influences nutrient cycling and decomposition processes (16). Predatory protists are a key link between the primary producers (such as microalgae) and higher trophic levels, including larger invertebrates and fish (17). Parasitic protists play an important role in maintaining ecosystem health by controlling dominant species, thus preventing any one species from monopolizing resources and promoting greater biodiversity (18, 19). Some protists can also serve as decomposers, playing a key role in nutrient cycling and energy flow (20, 21). Given the significance of benthic microeukaryotes in intertidal ecosystems, it is important to understand their community composition and biogeographic patterns for predicting ecosystem function as global environments change.

There have been a number of recent studies on intertidal microeukaryotic communities that have significantly advanced our understanding of their composition, distribution, and factors that affect them. For instance, Kong et al. (21) showed that differences between planktonic and benthic microeukaryotes were driven by environmental factors. Similarly, Pan et al. (22) highlighted the influence of environmental and spatial factors on the community structure, noting a significant correlation between community composition and factors such as temperature, salinity, and nutrients. Zhang et al. (23) extended these findings by investigating the biogeography of microeukaryotic communities in intertidal sandy beaches, showing there were similar geographical patterns in abundant and rare sub-communities and emphasized the role of environmental factors over spatial factors. Kalu et al. (24) also contributed to this understanding by investigating the dynamics of benthic protists in systems with very large tidal ranges, noting that stochastic processes, particularly dispersal limitation, significantly influenced community assembly. However, few recent studies have investigated seasonal variations in intertidal microeukaryotic communities (21, 22, 24). Seasonal changes are crucial as they can significantly influence community structure, function, and overall ecosystem processes due to variations in environmental conditions such as temperature, light, and nutrient concentrations (22, 25). Understanding these dynamics is vital for predicting how these ecosystems might respond to environmental changes, including climate change. The impact of sediment type (sandy or muddy) on microeukaryotic communities in intertidal sediments also remains under-explored. Sediment characteristics can influence microbial habitats, nutrient availability, and the physical environment, thus potentially shaping the community composition and biodiversity (22, 26). Seasonal dynamics of trophic functional groups (i.e., autotrophs, heterotrophs, and mixotrophs) in intertidal sediments have also not received sufficient attention. Understanding seasonal variations in the abundance and activity of these groups helps explain how intertidal ecosystem functioning changes throughout the year (27). These dynamics are key to understanding nutrient cycling, energy flow, and organic matter decomposition, which are essential processes that sustain these ecosystems (28).

Monthly samplings of intertidal surface sediments were conducted over a year. The investigation spanned two types of sediments (mud and sand) from two sampling stations in Qingdao, a representative area for northern China’s coastal ecosystems. Analysis of the eukaryotic community structure was performed using 18S rRNA sequencing, with a focus on alpha-diversity, operational taxonomic unit (OTU) composition, community composition, and network structure.

## MATERIALS AND METHODS

### Field sampling

**Fig 1:**
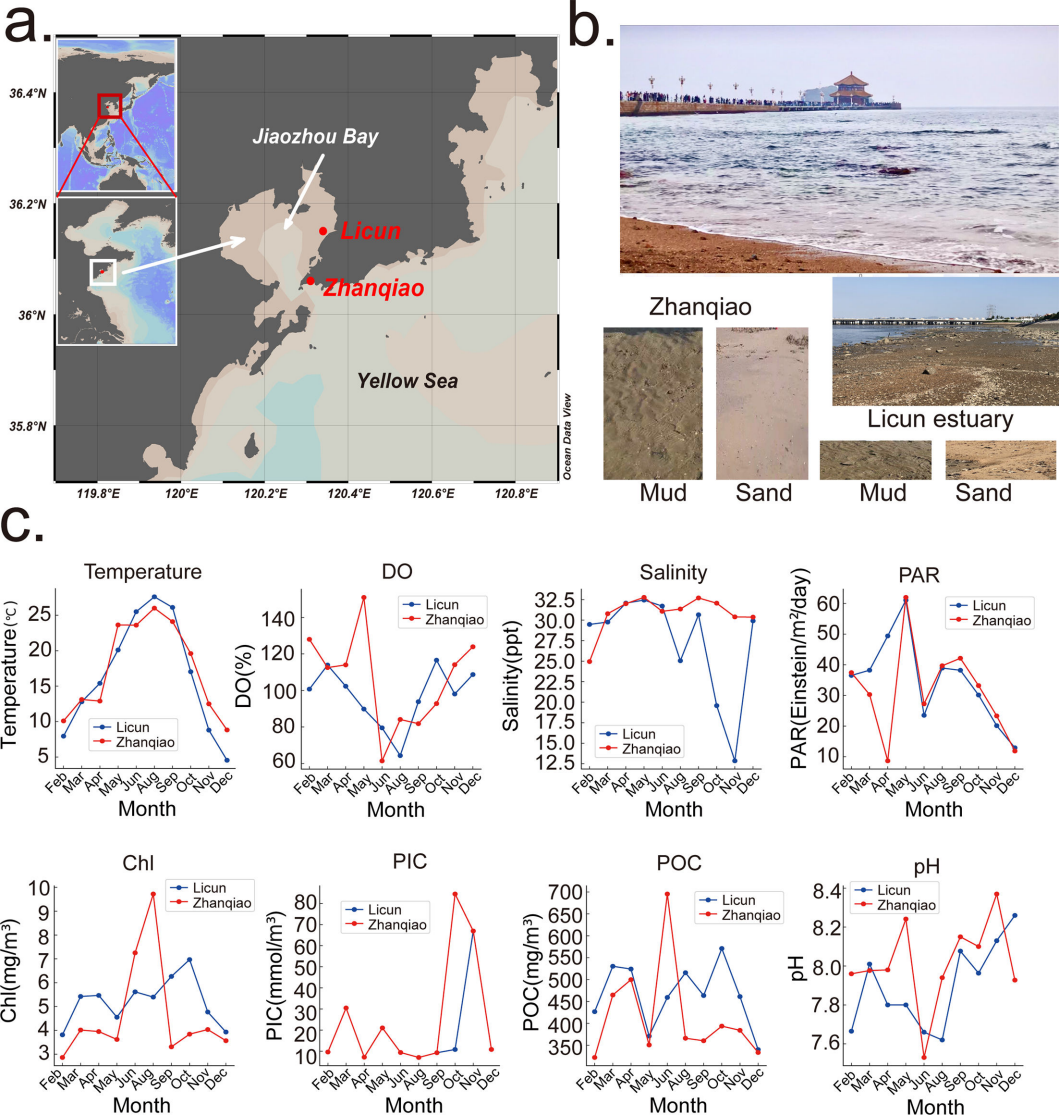
Sampling design. (**a**) Geographical information of the two stations: Field sampling was conducted in two intertidal zones of Qingdao, China. The sampling stations were at Licun Estuary (36.15 N, 120.34 E) and Zhanqiao Beach (36.06 N, 120.31 E). (**b**) Both muddy and sandy samples (top 2–5 mm) were collected during low tide (water levels < 90 cm). (**c**) Changes in eight environmental factors across 10 months at the two sampling stations. The eight environmental factors are temperature, DO, salinity, PAR, chlorophyll (Chl), pH, PIC, and POC. Environmental data were acquired either using a handheld multiparameter meter or through remote sensing data.

Field sampling was conducted at two intertidal zone sites in Qingdao, China. The sampling stations were at Licun Estuary (36.15 N, 120.34 E) and Zhanqiao Beach (36.06 N, 120.31 E), which are respectively situated inside and outside of Jiaozhou Bay (Fig. 1a). Sampling was carried out monthly for over 10 months. Both muddy and sandy samples (top 2–5 mm) were collected during low tide (water levels < 90 cm), around noon and in the afternoon (Fig. 1b). Samples were collected from near the water’s’ edge, where sediments were less exposed to air, minimizing moisture loss and additional stress. A total of 3 kg of sediment (1.5 kg mud and 1.5 kg sand) was collected from each station each time and subdivided into 50 mL collection tubes, which were then stored at −80°C. One liter of overlying seawater was collected at each station, and the seawater properties, including water temperature, dissolved oxygen (DO), total dissolved solids, salinity, and pH, were analyzed on site by a Pro Plus handheld multiparameter meter (YSI, USA). Remote sensing data from MODIS-Aqua were used to extract values for photosynthetically active radiation (PAR), chlorophyll (Chl), particulate inorganic carbon (PIC), and particulate organic carbon (POC). If daily data were not available, 8-day averages or monthly averages were used. The environmental data are shown in Fig. 1c.

### 18S rRNA gene sequencing

High-throughput sequencing of the 18S rRNA gene was used to investigate the responses of intertidal microeukaryotic communities to changes in season and sediment type. For each sediment type in each month, 10 g of sediment was sent to Majorbio (Shanghai, China) for DNA extraction, polymerase chain reaction (PCR), and high-throughput sequencing. DNA extraction was performed using the genomic DNA of the microbial community according to the instructions of the E.Z.N.A. Soil DNA Kit (Omega Bio-tek, Norcross, GA, USA). The quality of DNA was assessed by 1% agarose gel electrophoresis, and the DNA concentration and purity were determined using NanoDrop2000 (Thermo Scientific, USA). In brief, the V2–V3 regions of the 18S rRNA gene were amplified with PCR primers 82F_516R under the following conditions: an initial denaturation at 95°C for 3 minutes, 32 cycles of 95°C for 30 seconds, 55°C for 30 seconds, and 72°C for 45 seconds, and a final elongation at 72°C for 10 minutes (29, 30). Sequencing of amplified 18S rRNA genes was performed on an Illumina MiSeq PE300 platform. The raw sequencing data have been deposited in the NCBI Sequence Read Archive, and the BioProject accession number is PRJNA1268665.

### Photosynthetic parameters

Photosynthetic parameters were assessed using a Phyto-PAM II Fluorometer (Walz, Germany). Prior to each measurement, a 25 g sediment sample was mixed with 20 mL of filtered seawater. Five milliliters of seawater from the water/sediment interface was then extracted and placed in a centrifuge tube for 1 h of dark adaptation. This 5 mL sample was vigorously shaken and then allowed to settle for approximately 10 seconds before taking the PAM measurements. The PAM methodology was based on McMinn et al. (31). The Phyto-PAM II utilizes five measuring light wavelengths (440, 480, 540, 590, and 625 nm) for simultaneous excitation and deconvolution of four algae groups: cyanobacteria, green algae, brown algae (including diatoms), and red algae. This study focused on brown microalgae in terms of maximum quantum yield of photosystem II (*F*_*v*_/*F*_*m*_), photosynthetic efficiency (*α*), maximum relative electron transport rate (rETR_max_), and photoadaptive index (*E*_*k*_). *F*_*v*_/*F*_*m*_ is calculated by the following formula: *F*_*v*_/*F*_*m*_ = (*F*_*m*_ – *F*_*o*_)/*F*_*m*_. *F*_*o*_ is the minimum fluorescence yield of dark-adapted samples when all PSII reaction centers are open. *F*_*m*_ is the maximum fluorescence yield when all PSII reaction centers are closed. *F*_*v*_/*F*_*m*_ often serves as an indicator of the health and efficiency of the photosynthetic apparatus in plants, algae, and cyanobacteria. Rapid light curves (RLCs) were constructed to calculate the other three parameters. A RLC involves eight consecutive 10-second intervals of actinic light of increasing intensity with an accompanying yield measurement at the end of each actinic interval; this plots the relative electron transport rate (rETR) against increasing light intensities (PAR) (32). The rETR was determined by multiplying the irradiance by the quantum yield measured at the end of that interval (33). RLCs were described using the model of Platt et al. (34) and various non-linear regression curve fitting techniques (35). The initial slope of the function is termed *α*, which measures algal cells’ ability to utilize light. As the function reaches a plateau, the rETR_max_ occurs. *E*_*k*_ is the photoadaptive index or light saturation parameter and is calculated by the equation: *E*_*k*_ = rETR_max_/*α* (36).

### Bioinformatics and statistical analysis

Quality control of the raw sequencing data was conducted using fastp (version 0.19.6) (37). Read merging was performed with FLASH (version 1.2.11) (38): (i) low-quality bases (average quality score < 20) within a 50 bp sliding window at the read tails were trimmed, reads shorter than 50 bp after trimming were discarded, and reads containing ambiguous bases (N) were removed; (ii); paired-end reads were merged based on a minimum overlap of 10 bp; (iii) sequences with an overlap mismatch rate exceeding 0.2 were filtered out. Barcode and primer sequences at the ends of the reads were removed. The merged sequences were clustered into OTUs at 97% similarity using UPARSE, and the most abundant sequence in each OTU was selected as the representative. Taxonomic annotation of representative sequences was performed using RDP Classifier with a confidence threshold of 0.70, based on the SILVA database (release 138.1/18s_eukaryota).

This study utilized 18S data to examine α-diversity, community composition, co-occurrence networks, and trophic functional groups in the intertidal microeukaryotic communities. These bioinformatics analyses were conducted by R software and the online platform of Majorbio Cloud Platform (www.majorbio.com), a comprehensive online platform for multiomic analyses (39). The Shannon index, Chao 1, and ACE were used to measure α-diversity (Mothur version 1.30.2). To assess differences in community composition, analysis of similarity (ANOSIM) and principal coordinates analysis (PCoA) were applied, based on Bray-Curtis dissimilarity using the R package Vegan (version 2.5-3). Linear discriminant analysis effect size (LEfSe) was used to identify key taxa that significantly differentiate groups (LDA > 2.0 and *P <* 0.05). In addition, key aspects of the co-occurrence network, such as nodes, edges, and average degree, were analyzed and visualized using the R package igraph (version 1.3.2). The Pearson correlation coefficient (*r* > 0.7 and *P* < 0.01) was calculated between two OTUs. The layout was calculated based on layout_with_fr. In each network diagram, the top 18 modules are given a different color. All OTUs were included in the correlation analysis. Canonical correspondence analysis (CCA) and the Mantel test were utilized to explore the relationships between community composition and environmental factors. Correlations between taxa and environmental factors were determined using Spearman correlation values. The classification of trophic functional groups of protists was based on the frameworks provided by Hörstmann et al. (40), Käse et al. (41), and Schneider et al. (42). Depending on the data type, significant differences between two groups were assessed using either the Wilcoxon rank-sum test or *t*-test, and among more than two groups using either the Kruskal-Wallis *H* test or ANOVA.

## RESULTS

### Response of microeukaryotic communities to changes in month and sediment type (α-diversity, community composition, and co-occurrence network)

**Fig 2:**
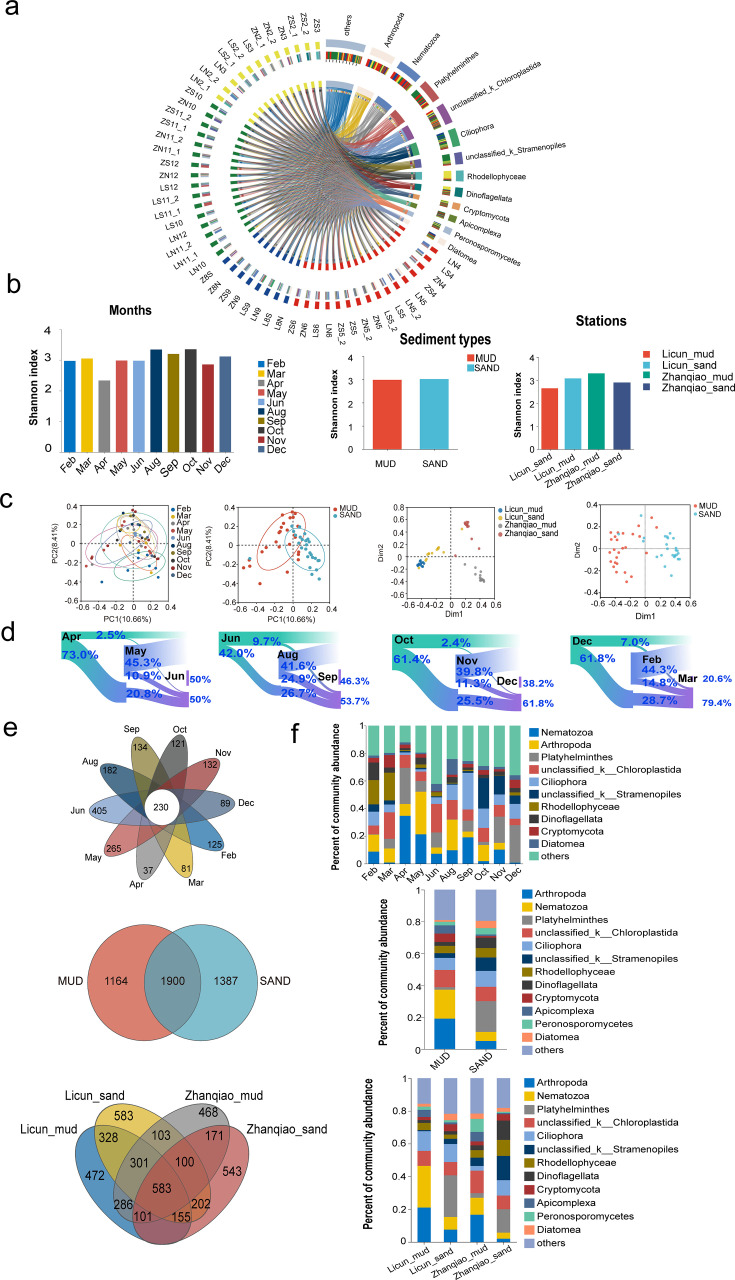
Alpha-diversity, beta-diversity, and relative abundance. (**a**) Microeukaryotic community composition across 10 months and 4 seasons. (**b**) Comparison of α-diversity of microeukaryotic communities between sediment type, station, and month (all *P* > 0.05). (**c**) PCoA and Random Forest plots showing the differences in microeukaryotic community across month, sediment type, and station (all *P* < 0.01). (**d**) Sankey diagram illustrating fluctuations in abundant OTUs across different months within four seasons. (**e**) The unique and shared OTUs in different months, sediment types, and stations. (**f**) Microeukaryotic community composition (phylum level) across month, sediment type, and station.

Based on 18S rRNA sequencing data, 3,535,713 high-quality sequences were obtained, comprising 1,578,043,525 bases with an average read length of 446 bp. A total of 4,452 OTUs were identified, including 80 phyla, 180 classes, 323 orders, 400 families, 718 genera, and 979 species. The responses of the microeukaryotic communities to changes in month and sediment type were analyzed by examining α-diversity, community composition, and co-occurrence networks. In total, 56 samples across 10 months in a single year were analyzed (Fig. 2a). The α-diversity showed no significant differences with month or sediment type and only a small, non-significant influence based on sampling site by Shannon index (months: Kruskal-Wallis *H* test, *P* = 0.917; sediment: Wilcoxon rank-sum test, *P* = 0.791; stations: Kruskal-Wallis H test, *P* = 0.224; Fig. 2b). Similarly, the Chao1 and ACE indices showed no significant variation across months but revealed significant differences between sediment types and among stations (Chao1: months: Kruskal-Wallis *H* test, *P* = 0.681; sediment: Wilcoxon rank-sum test, *P* = 0.0009; stations: Kruskal-Wallis *H* test, *P* = 0.0062. ACE: months: Kruskal-Wallis *H* test, *P* = 0.710; sediment: Wilcoxon rank-sum test, *P* = 0.0004; stations: Kruskal-Wallis *H* test, *P* = 0.0032; Fig. S1). However, the community composition was significantly affected by month and sediment type (ANOSIM, P_months_ < 0.01, P_sediment_ < 0.01; Fig. 2c). Sankey diagrams show fluctuations in abundant OTUs (exceeding 0.1%) across different months. Minimal variation occurred between April and May, and maximal changes were evident from May to June (Fig. 2d). June had the highest number of OTUs (405), while April had the lowest number (40) (Fig. 2e). Although numerous OTUs (1,900) were shared between mud and sand, there were also some abundant OTUs that were specific to one substrate (mud: 1,164 and sand: 1,387), with distinct OTU compositions occurring in the mud and sand across locations (Fig. 2e).

**Fig 3:**
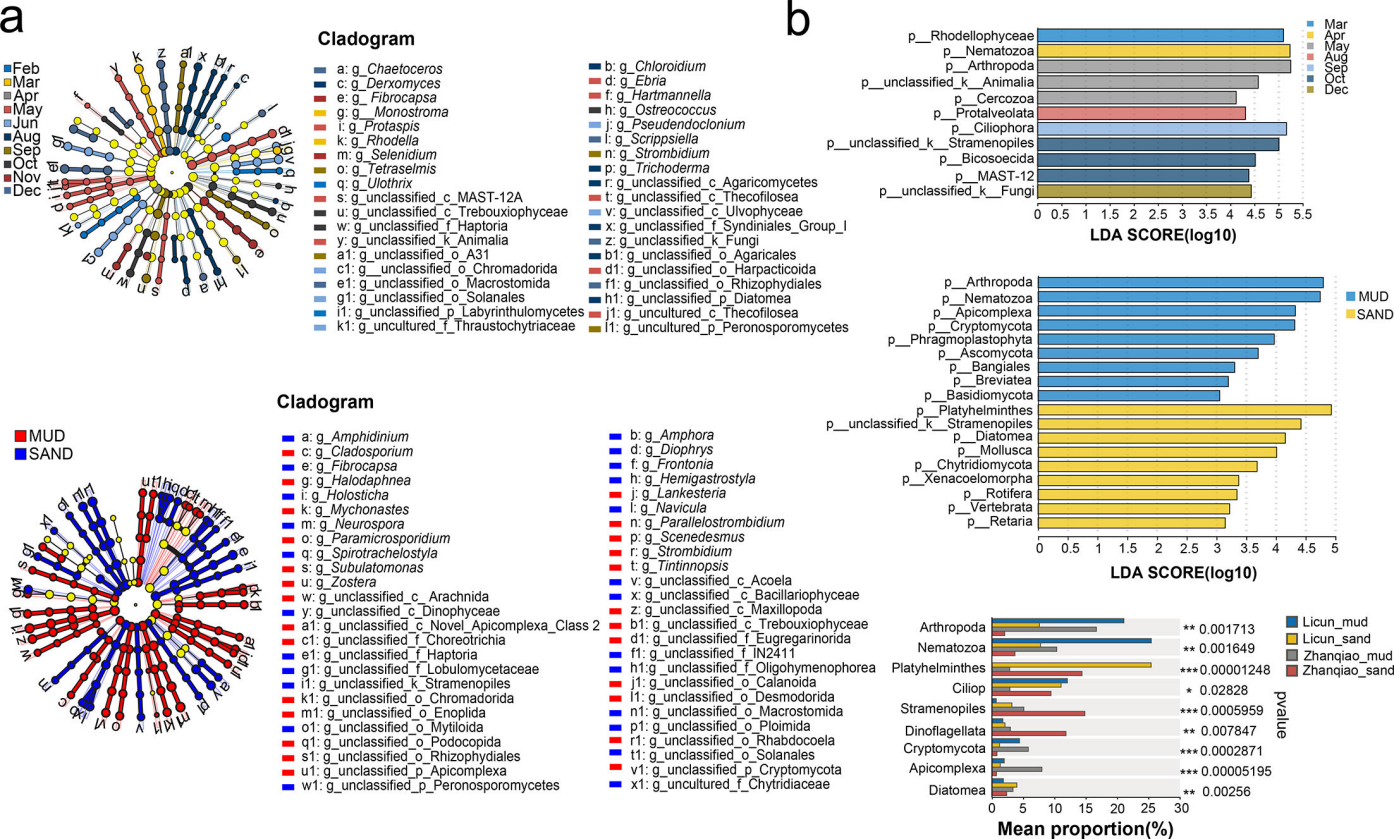
LEfSe analysis of time series and two types of sediment between two stations. (**a**) Genera that exhibited significant variations across different months and sediment types. (**b**) Key phyla that significantly differentiate months, sediment types, and stations.

The dominant phyla (top 10 phyla) were similar for all months, but the relative abundance differed even between adjacent months (Fig. 2f). The abundance of Nematozoa, Arthropoda, Ciliophora, Stramenopiles, and Rhodellophyceae differed significantly between months (Kruskal-Wallis *H* test, *P*_Nematozoa_ < 0.05, *P*_Arthropoda_ < 0.05, *P*_Ciliophora_ < 0.05, *P*_Stramenopiles_ < 0.05, and *P*_Rhodellophyceae_ < 0.01; Fig. 2f). Nematozoa abundance peaked in April, Arthropoda in May, Ciliophora in September, Stramenopiles in October, and Rhodophyceae in February (Fig. 2f). Thirty-eight genera exhibited significant differences between months (LDA > 3, Fig. 3a). The dominant phyla in mud and sand in each month were similar, but the relative abundance differed between mud and sand (Fig. 2f). The abundance of Arthropoda, Nematozoa, Platyhelminthes, Stramenopiles, Cryptomycota, and Apicomplexa differed significantly between mud and sand (Wilcoxon rank-sum test, *P*_Arthropoda_ < 0.01, *P*_Nematozoa_ < 0.01, *P*_Platyhelminthes_ < 0.01, *P*_Stramenopiles_ < 0.01, *P*_Cryptomycota_ < 0.01, and *P*_Apicomplexa_ < 0.01; Fig. 2g). In addition, 50 genera showed differences between mud and sand (LDA > 3, Fig. 3a). Further analysis by location found that Arthropoda and Nematozoa were most abundant in the mud from Licun, Platyhelminthes, and Diatomea in the sand from Licun, Stramenopiles, and Dinoflagellata in the sand from Zhanqiao, and Cryptomycota and Apicomplexa in the mud from Zhanqiao (Fig. 3b).

**Fig 4:**
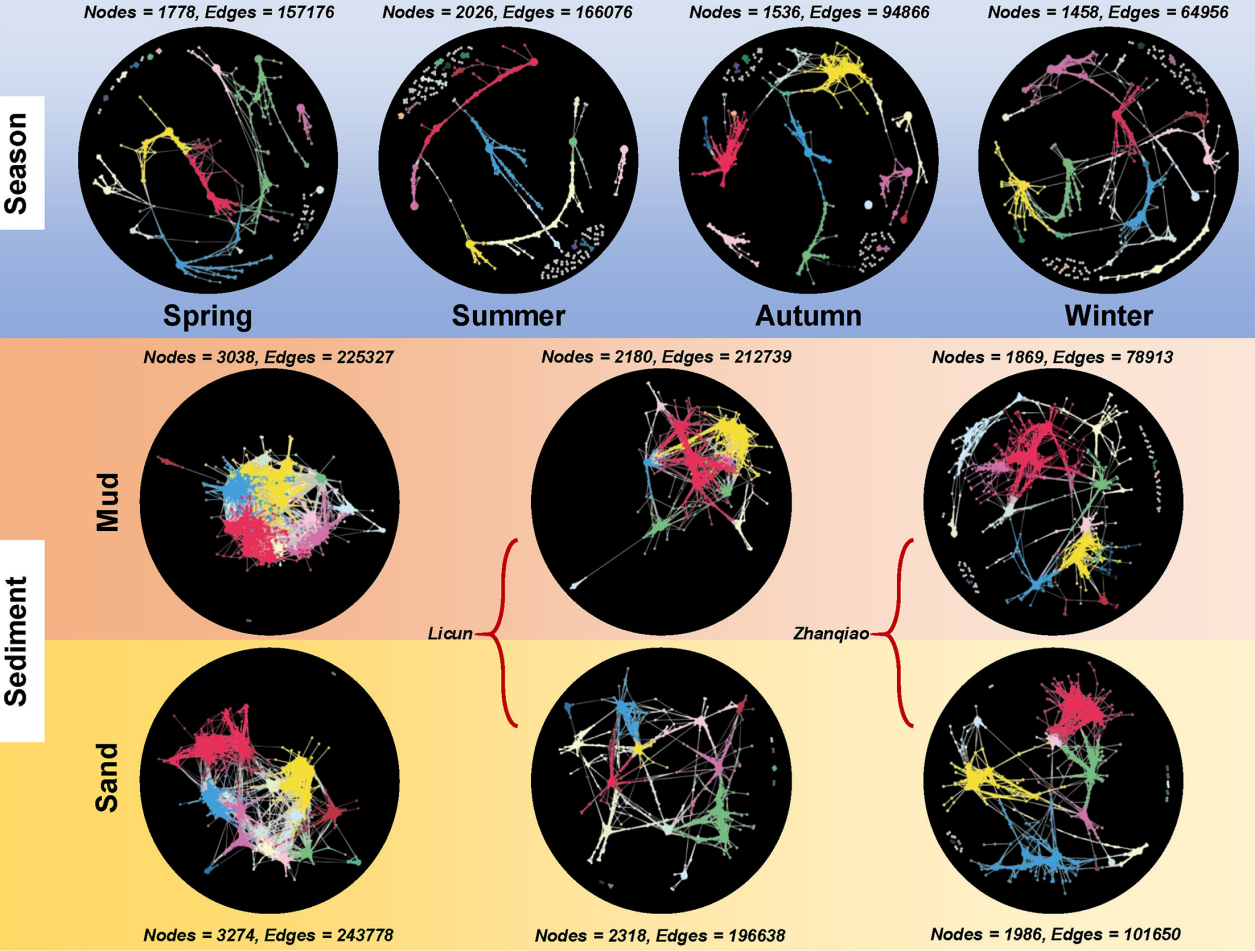
Co-occurrence network of the microeukaryotic community across different seasons, sediment types, and stations.

The response of co-occurrence networks within microeukaryotic communities to changes in season and sediment types was analyzed (Fig. 4). Seasonal analysis showed an initial increase in interactions from spring (157,176 edges, average degree 176.80) to summer (166,076 edges, average degree 163.94), followed by a decline in both network size and density through autumn (94,866 edges, average degree 123.52) and winter (64,956 edges, average degree 89.10), illustrating a clear seasonal fluctuation in network dynamics (Fig. 4). Comparing mud and sand, the former exhibited 225,327 edges with an average degree of 148.34, versus sand’s 243,778 edges and an average degree of 148.92, indicating comparable interaction densities (Fig. 4). Further analysis by location found that the Licun mud network (212,739 edges, average degree of 195.17) was denser than its sand counterpart (196,638 edges, average degree of 169.66), while the Zhanqiao network showed the opposite pattern (sand: 101,650 edges, average degree of 102.37; mud: 78,913 edges, average degree of 84.44).

### Environmental factors of different months and sediments

**Fig 5:**
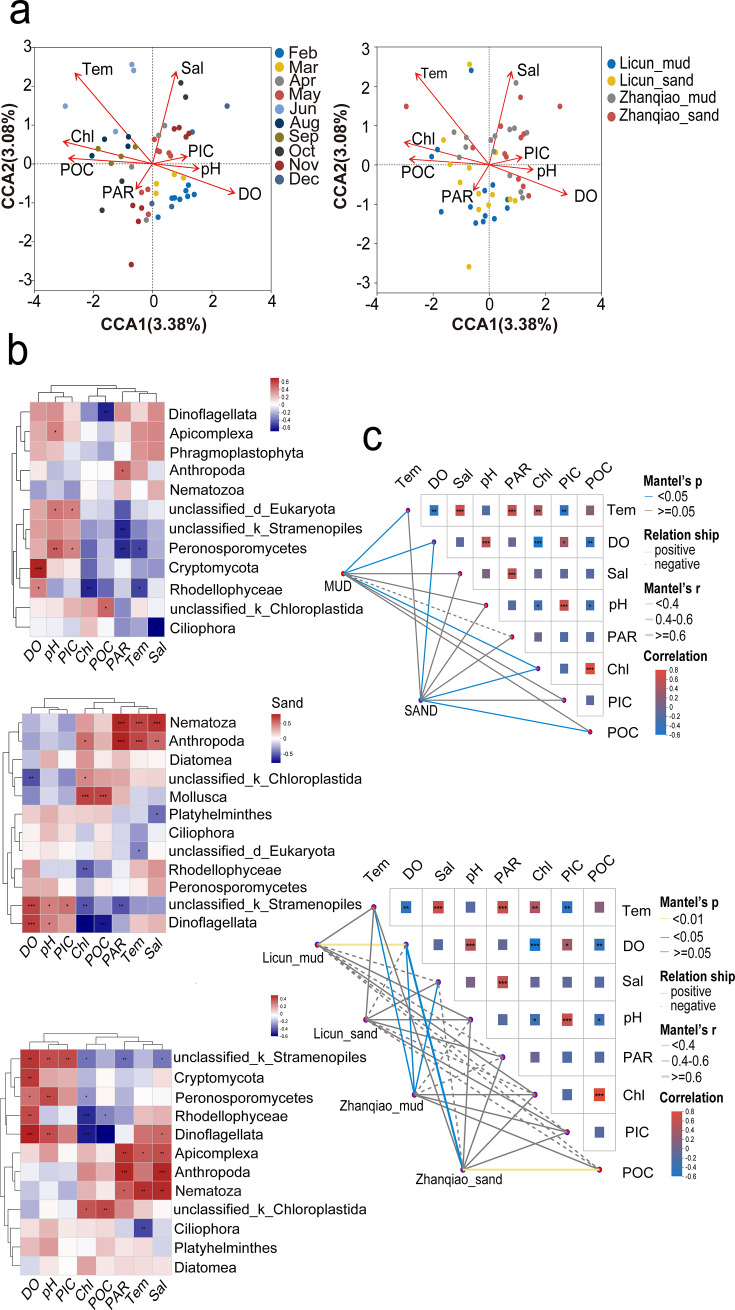
Environmental factor analysis. (**a**) CCA plot showing the correlation between community composition and environmental factors (*P* < 0.01). (**b**) Correlations between taxa (phylum level) and environmental factors. *P < 0.05, **P < 0.01, and ***P < 0.001. (**c**) Mantel test heatmap illustrating the correlation between community composition and environmental factors.

Microeukaryotic community structures in intertidal sediments are significantly influenced by environmental factors, as evidenced by the CCA, which identified temperature, DO, salinity, chlorophyll *a* (chl *a*), pH, and POC as key factors (permutest, *P*_temp_ < 0.01, *P*_DO_ < 0.01, *P*_sal_ < 0.01, *P*_pH_ < 0.05, *P*_chl_ < 0.01, and *P*_POC_ < 0.01; Fig. 5a). Stramenopiles, Peronosporomycetes, and Dinoflagellata were positively correlated with DO and pH but negatively correlated with chl *a*. Apicomplexa, Arthropoda, and Nematozoa were positively correlated with temperature and PAR (*P* < 0.05, Fig. 5b).

Mantel tests show significant correlations between communities and temperature, DO, and chl *a* in mud environments, and DO, chl *a*, and POC in sand environments (*P* < 0.05, Fig. 5c). In mud environments, Peronosporomycetes showed positive correlations with pH and PIC, but negative correlations with PAR and temperature. Rhodellophyceae and Cryptomycota were positively associated with DO, with Rhodellophyceae also negatively correlated with chl *a* and temperature (*P* < 0.05, Fig. 5b). In sand environments, Nematozoa and Arthropoda exhibited positive correlations with temperature, salinity, and PAR. Stramenopiles and Dinoflagellata were positively correlated with DO and pH, but negatively correlated with chl *a* (*P* < 0.05, Fig. 5b). Subsequent location-based analysis identified significant correlations between eukaryotic communities and DO in the Licun mud (*P* < 0.01), and temperature, DO, and salinity in the Zhanqiao mud (*P*_temp_ < 0.05, *P*_DO_ < 0.05, and *P*_sal_ < 0.05), and DO and POC in the Zhanqiao sand (*P*_DO_ < 0.05 and *P*_POC_ < 0.01; Fig. 5c).

### Response of autotrophs, heterotrophs, and mixotrophs and photosynthetic parameters to month and sediment type

**Fig 6:**
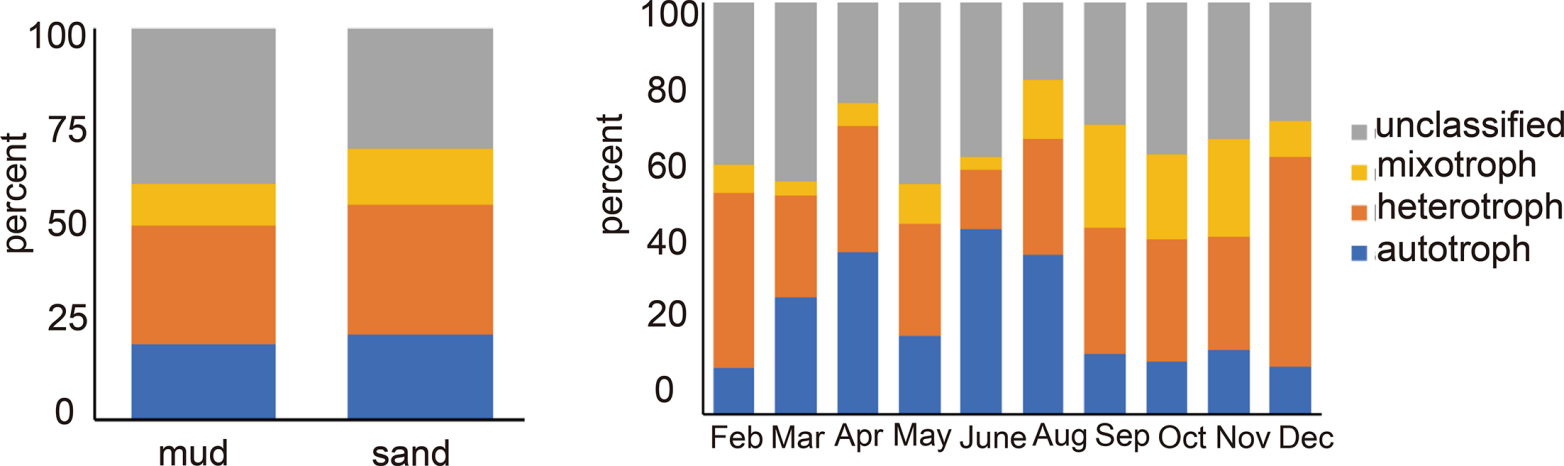
The relative abundance of autotrophs, heterotrophs, and mixotrophs across different months and sediment types.

The abundance of autotrophs, heterotrophs, and mixotrophs in the protist communities was analyzed with regard to month and sediment type. The classification of trophic functional groups of protists was based on the frameworks provided by Hörstmann et al. (40), Käse et al. (41), and Schneider et al. (42). Sediment type did not significantly affect their abundance (Wilcoxon rank-sum test: *P*_autotroph_ = 0.332, *P*_heterotroph_ = 0.621, and *P*_mixotroph_ = 0.360; Fig. 6). Monthly changes influenced all three groups, with heterotrophs showing the largest, although non-significant, changes (Kruskal-Wallis *H* test, *P*_autotroph_ = 0.088, *P*_heterotroph_ = 0.050, and *P*_mixotroph_ = 0.065; Fig. 6). Peak abundances of autotrophs were found in June, heterotrophs in December, and mixotrophs from September to November (Fig. 6).

**Fig 7:**
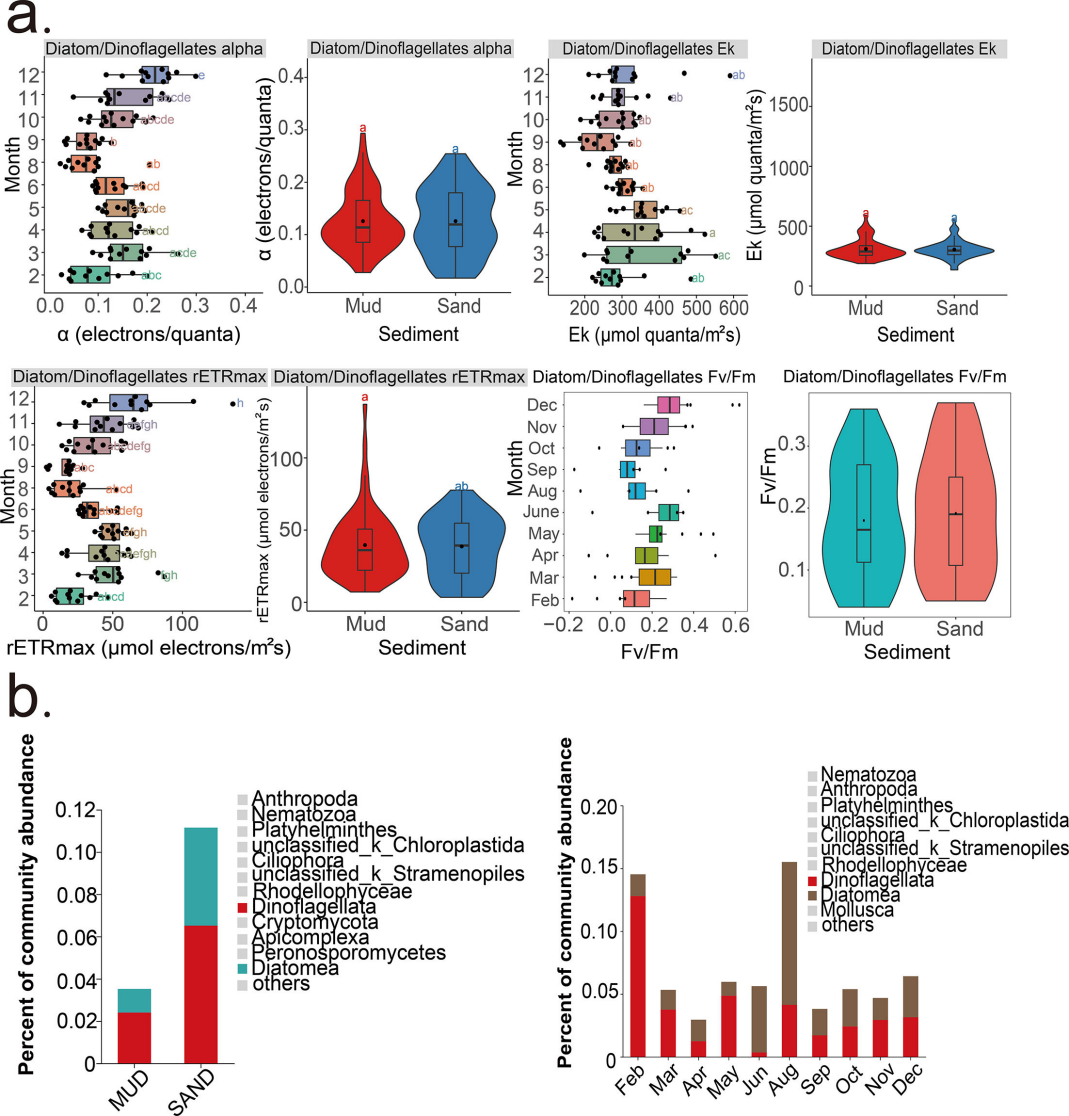
The photosynthetic parameters of diatoms/dinoflagellates. Statistically significant differences were analyzed by ANOVA tests. (**a**) The *α*, rETR_max_, *E*_*k*_, and *F_v_*/*F_m_* of microalgae across different months and sediment types. (**b**) The relative abundances of diatoms and dinoflagellates across different sediment types and months.

The photosynthetic efficiency (*α*), maximum relative electron transport rate (rETR_max_), the maximum quantum yield (*F_v_*/*F*_*m*_), and photo adaptive index (*E*_*k*_) of microalgae, specifically diatoms and dinoflagellates (brown group in PAM analyses), were analyzed in relation to changes in month and sediment type. The *α*, rETR_max_, *F_v_*/*F_m_*, and *E*_*k*_ values in the mud and sand were similar (ANOVA, *P*_α_ =0.997, *P*_rETRmax_ = 0.811, *P*_Ek_ = 0.715; *P*_*Fv*/*Fm*_ = 0.528; Fig. 7a), but varied between months (ANOVA, *P*_α_ < 0.01, *P*_rETRmax_ < 0.01, *P*_Ek_ < 0.01; *P*_*Fv/Fm*_ < 0.01; Fig. 7a). The *α* and rETR_max_ values of these microalgae peaked in December and were lowest in February, August, and September. The *E*_k_ value was highest in March, April, and May and lowest in September. The *F_v_*/*F_m_* value was highest in March, June, and December and lowest in September. Interestingly, diatoms and dinoflagellates were more abundant in February and August, despite their lower *α* and rETR_max_ values during these months (Fig. 7b).

## DISCUSSION

### Change in microeukaryotic communities across months

This study assessed the responses of microeukaryotic communities to changes in seasons in terms of α-diversity, OTU composition, community composition, and co-occurrence network. The α-diversity was not significantly influenced by the month, as determined by the Chao1, ACE, and Shannon indices. This is consistent with the results of Kong et al. (21), who likewise did not notice statistical differences in intertidal eukaryotic communities between seasons. The reason for this stable α-diversity may be that these microeukaryotic communities have adapted to be resilient to environmental stressors, and this resilience allows them to maintain community structure and function across different timescales (43). It is also possible that local biogeographic factors and ecological interactions (such as competition and predation) could buffer the communities against temporal changes (44). The composition of abundant OTUs (exceeding 0.1%) fluctuated between months, with June having the highest number and April the lowest. This is the first time that fluctuations in microeukaryotic OTU composition in intertidal sediments across months have been reported. The high number of OTUs reported in June is possibly because of the extended daylight hours and increased temperatures in this month that might have increased the growth rate of the photosynthetic and thermophilic organisms in the intertidal zone (21). Many eukaryotic taxa in intertidal zones have fixed breeding seasons. For example, some algae and marine invertebrates may have peak reproductive activity in early summer (June), resulting in increased detection of their genetic material in intertidal samples (45). However, the reason for the lowest number of OTUs in April is unclear.

The community composition of microeukaryotes in intertidal sediments varied significantly by month, aligning with previous observations of seasonal fluctuations (9, 21, 24). According to the CCA, the monthly changes in community composition were closely linked to changing environmental conditions, including factors such as temperature, dissolved oxygen, and salinity. Temperature naturally increases from spring to summer and then decreases in autumn and winter, influencing the growth, survival, and feeding of eukaryotes, and thus controlling their densities and community composition in intertidal sediments (10, 46). DO can fluctuate with temperature, with warmer waters typically containing less oxygen. Dissolved oxygen is essential for aerobic respiration in marine organisms, and variations in DO levels can impact the survival, growth, and distribution of eukaryotic communities in intertidal zones (47). Salinity can vary due to precipitation, evaporation, and freshwater influx, which are also often seasonal. Salinity influences the osmoregulation processes of marine organisms and can affect species distribution and community diversity (48). Microeukaryotes have varying tolerances to salinity changes, and this can lead to shifts in community structure as some species may be more competitive or adaptable to changes in salinity (49, 50).

In this study, Nematozoa, Arthropoda, Platyhelminthes, unclassified_k_Chloroplastida, Ciliophora, unclassified_k_Stramenopiles, Rhodellophyceae, Dinoflagellata, Crytomycota, and Diatomea had a high relative abundance of the sequences. It is noted that the relative abundance of microeukaryotes inferred from 18S rRNA gene sequencing may be either greater or lower than their actual abundance, because eukaryotic genomes can contain multiple copies of this gene (39, 51). Therefore, changes observed in the proportion of gene reads need to be interpreted with caution. In this study, the abundance of Ciliophora, Stramenopiles, and Rhodellophyceae was found to significantly differ between months, with Ciliophora peaking in September, Stramenopiles in October, and Rhodophyceae in February. The high abundance of Ciliophora in September may be attributed to favorable environmental conditions and food availability (52, 53). September often marks the transition between summer and autumn, providing moderate temperatures and stable conditions that are favorable for Ciliophora (52, 54). The abundance of Ciliophora is also likely influenced by the availability of food resources, such as bacteria and other microorganisms, which might be particularly plentiful during this month due to organic matter accumulation from earlier months (53). The high abundance of Stramenopiles in October may also be due to post-summer nutrient availability. Like September, October represents a transitional period with moderate temperatures and sufficient light that can support the growth and reproductive cycles of Stramenopiles (55). Furthermore, after summer, the decomposition of organic matter increases nutrient availability in the sediments, providing a nutrient-rich environment for Stramenopiles (56). The peak abundance of Rhodophyceae in February is likely due to reduced competition and predation, coupled with more favorable light conditions during this month. During February, the weather is still cold, and this might reduce competition and predation from other marine organisms, allowing Rhodophyceae to thrive (57). Furthermore, light conditions in February, before the onset of longer spring days, may be optimal for the photosynthesis of Rhodophyceae. Rhodophyceae are typically characteristic of lower light conditions, and this also might contribute to their enhanced abundance (58–60).

This is the first study to explore the responses of co-occurrence networks within intertidal microeukaryotic communities to seasonal changes over a whole year. It was found that the number of interactions and the density of the network increased from spring to summer and then decreased through autumn and winter, showing a pronounced seasonal fluctuation in network dynamics. The increase in the number of interactions and network density in spring and summer can be explained by enhanced biological activity and nutrient availability during this period. Warmer temperatures and longer daylight hours in spring and summer promoted metabolic activity and growth rates among microeukaryotic organisms, leading to more interactions (61). This can include competition, predation, symbiosis, and other ecological interactions as organisms become more active and abundant. As primary producers thrive, so do the consumers and decomposers in the network, increasing the overall interactions among community members (62). During autumn and winter, however, as temperatures drop and daylight hours shorten, the growth rates and metabolic activities of many eukaryotic organisms decrease, leading to lower primary production, reduced reproduction, and fewer interactions (63).

### Differences between microeukaryotic communities in mud and sand

This study also investigated the differences between microeukaryotic communities from muddy and sandy sediments using 18S rRNA analysis (21, 24). The differences in the communities were assessed by examining α-diversity, OTU composition, community composition, and co-occurrence networks. The α-diversity results showed that the α-diversity of microeukaryotic communities was not significantly different between sand and mud based on the Shannon index but was significantly different according to the Chao1 and ACE richness estimators. This suggests that there is a significant difference in the potential species richness of microeukaryotic communities between muddy and sandy environments, which may be attributed to factors such as sediment grain size, porosity, water retention capacity, and organic matter content. These environmental characteristics can influence the habitat suitability for certain microorganisms. Therefore, the results indicate that different sediment types significantly affect the species richness of microeukaryotic communities, while having a relatively minor impact on the evenness of their community structure (22, 64). In contrast, this similarity in Shannon diversity may be because both sandy and muddy substrates perform similar ecological functions that support a diverse range of microeukaryotic organisms (65). Factors such as organic matter content, oxygen availability, and microbial activity could be similarly conducive to biodiversity in both types of sediment. For OTU composition, it was found that there were many OTUs (1,900) common to both mud and sand substrates, with a significant number found only in one type (1,164 in mud and 1,387 in sand). The existence of these common OTUs is likely because both mud and sand substrates share overlapping environmental conditions, such as salinity, temperature, and exposure to tidal influences, supporting the survival and growth of some microeukaryotic OTUs across both substrate types (21). The reason why some OTUs only occurred in one sediment type may be because mud and sand substrates differ significantly in their physical properties, such as grain size, porosity, and organic content, and these affect the availability of nutrients and habitat niches (64). These differences may promote the development of specific communities adapted to the differing conditions of each substrate.

The community composition of microeukaryotes in intertidal sediments differed significantly between mud and sand substrates. The primary cause of this difference is likely to be that mud and sand substrates have significantly different grain sizes. Grain size has a significant effect on the variation of community composition in intertidal sediments (21, 22, 66). Variations in grain size affect oxygen availability, moisture retention, and the presence of deposit-feeding macrofauna, all of which influence the biological communities within these sediments (22, 64). Also, the organic content is typically higher in mud than in sand, providing more nutrients to the microbial and microeukaryotic communities (67). This difference in nutrient availability can lead to distinct communities, as some microeukaryotes are more adapted to nutrient-rich environments than others (65). The Mantel test results show that temperature, dissolved oxygen, and chlorophyll *a* are crucial in shaping the eukaryotic community in muddy intertidal environments. In sandy environments, DO, chl *a*, and POC are pivotal factors in influencing community dynamics. It is possible to suggest that microeukaryotic communities in muddy intertidal sediments are potentially vulnerable to ocean warming and hypoxia, while those in sandy sediments are more susceptible to hypoxia and pollution. These results underscore the potential vulnerability of sediment-based ecosystems to shifts in temperature, oxygen levels, and pollution.

Stramenopiles were notably more abundant in sand than mud, while Cryptomycota and Apicomplexa were more abundant in mud than in sand. Stramenopiles may preferentially thrive in sandy substrates due to their ability to adhere to sand grains and exploit the rapid drainage and oxygenation that sandy sediments typically provide (68). Cryptomycota, which are fungal-like organisms requiring moist conditions, favor muddy substrates as they have higher organic content and greater moisture retention (69). Apicomplexa, which are often parasitic, benefit from the higher density of potential hosts in nutrient-rich muddy environments (24). Additionally, this study found that the dominant phyla in both mud and sand varied by location, underscoring that geographical distance or dispersal limitations can significantly influence community composition in intertidal sediments (21). The Mantel test results show that in mud environments, Peronosporomycetes are positively correlated with pH and negatively with temperature, and Rhodellophyceae are positively linked to DO and negatively to temperature. In sand, both Stramenopiles and Dinoflagellata positively correlated with DO and pH. This suggests that certain common phyla in intertidal sediments may be susceptible to ocean warming, acidification, or hypoxia.

The co-occurrence network analysis showed that the number of interactions and network density are similar between mud and sand. This similarity is possible because both sediment types, despite their different physical properties, might support similar ecological processes such as decomposition, nutrient cycling, and microbial interactions (43). These processes are essential for sustaining a range of life forms, thereby fostering a similar number of interactions across different sediment types. Furthermore, despite the differences in texture and porosity between mud and sand, similar biotic factors, such as taxonomic composition, and abiotic factors, such as temperature and salinity, are similar across substrates, and this can lead to similar ecological network structures (70). Further network analysis by location revealed that the Licun (sampling site 1) mud network was denser than its sand counterpart, while the Zhanqiao (sampling site 2) network showed the opposite pattern. This opposite pattern may be attributed to differences in local water chemistry, sediment composition, and hydrodynamic conditions between the two sites (22, 61). In addition, the inherent biological characteristics of the communities at each site, such as species composition and functional traits, could drive differences in network density (71).

### Autotrophy, heterotrophy, and mixotrophy in microeukaryotic communities: abundance and photosynthetic parameters of brown microalgae

Sediment type did not significantly impact the relative abundance of these three trophic functional groups: autotrophs, heterotrophs, and mixotrophs. This may reflect a high degree of functional redundancy within these trophic groups across sediment types (72). This means that different taxa within the same trophic group might perform similar ecological roles, thus ensuring stable functional group abundance despite changes in sediment type. Also, both muddy and sandy sediments can provide sufficient resources to support these functional groups. The critical factors influencing their abundance might not be sediment type but other environmental conditions, such as light, temperature, or hydrodynamic conditions, which might have been similar at the studied muddy and sandy sites (61). Furthermore, the results showed that heterotrophs were more diverse and more abundant than autotrophs and mixotrophs in intertidal sediments, which is consistent with the results of Kalu et al. (24). The greater diversity of heterotrophs can probably be attributed to the organic matter availability in intertidal environments, which provides ample resources for heterotrophic organisms (73). However, it is important to note that many OTUs in the samples have not yet been categorized into a trophic functional group.

Unlike the sediment type, monthly changes influenced all three trophic functional groups. Peak abundances were recorded for autotrophy in June, heterotrophy in December, and mixotrophy between September and November. June typically marks the onset of summer in the northern hemisphere, providing elevated light and temperatures for photosynthesis (74). Sediments in December, the start of winter, are often characterized by abundant detritus and organic material from decaying organisms, which provide a rich source of nutrients for heterotrophs in this month (75). The autumn months (September and November) represent a transitional period that supports mixotrophs that can utilize both photosynthesis and heterotrophy (76). These months typically still have sufficient light for photosynthesis, while the increase in organic material makes them optimal for mixotrophs (77).

This study further examined how “brown microalgae,” including diatoms and dinoflagellates, reacted to monthly fluctuations and different sediment types, focusing on their abundance and photosynthetic parameters (*α*, rETR_max_, *E*_*k*_, and *F_v_*/*F*_*m*_). Alpha (*α*) represents the photosynthetic efficiency of photosystem II. rETR_max_ is a measure of the maximum relative electron transfer rate between photosystem II and photosystem I and is often used as a proxy for maximum photosynthetic capacity (32). *F_v_*/*F_m_* is a commonly used measure of the maximum capacity of the photosystem II in converting light energy into charged states and transferring it to the photochemical pathway. It represents a sensitive indicator of photosynthetic stress (31). *E*_*k*_, the photoacclimation index, is a measure of how well the photosynthetic apparatus is acclimated to ambient light. In well-acclimated systems, the *E*_*k*_ value will be approximately the same as the ambient light level (31). The high *α*, rETR_max_, and *F_v_*/*F_m_* values in December may result from evolutionary adaptations that maximize photosynthetic output under limited light conditions in winter (36). The low *α*, *F_v_*/*F_m_*, and rETR_max_ values in February are linked to reduced light availability and cold temperatures, while in August, they are associated with high temperatures and excessive light irradiance (78). Ambient PAR was obtained from satellite measurements and is shown in Fig. 1. The data show that PAR was highest in May and lowest in December. The high *E*_*k*_ values observed in March, April, and May reflect the increasing daylight during these months. These *E*_*k*_ values are correlated with the ambient PAR values, indicating that the autotrophs were able to adapt to seasonal increases in light. The microalgae acclimate to higher light intensities by raising their *E*_*k*_ values, thereby optimizing their photosynthetic apparatus to efficiently utilize the abundant light (36, 79). However, despite having the longest daylight, the *E*_*k*_ value and PAR in June were not the highest. It is likely due to photoinhibition caused by excessively high light intensities, which can damage the photosynthetic apparatus (79). Additionally, it is possible that elevated temperatures in June can induce thermal stress, further reducing photosynthetic efficiency and lowering *E*_*k*_ values (80).

The high abundance of brown microalgae in February and August, despite low *α*, *F_v_*/*F_m_*, and rETR_max_ values during these months, can be attributed to their fast growth rates. Diatoms and other brown microalgae are known for their rapid growth rates (81). They can undergo quick cycles of division when conditions are briefly optimal or when they have accumulated enough resources during less favorable conditions (81, 82). This ability allows them to maintain or increase their population size even when photosynthetic efficiency is not at its peak. The relative abundance of diatoms peaked in August, while dinoflagellates peaked in February. Diatoms apparently preferred the environmental conditions, such as sufficient sunlight and stable water temperatures, which are conducive to photosynthesis and nutrient uptake (83). Dinoflagellates seemed to do better in colder temperatures and benefited from nutrient-recycling processes that occur in winter. These nutrients become available due to the decomposition of organic matter and can be less competitively sought after during colder months (84).

Regarding sediment type, *α*, rETR_max_, *F_v_*/*F_m_*, and *E*_*k*_ values of brown microalgae in mud and sand are similar. It is likely because both mud and sand in intertidal zones experience similar light regimes due to periodic exposure to sunlight during low tide and submersion during high tide. This results in comparable light environments for microalgae in both sediment types, leading to similar photosynthetic parameters (85, 86). Additionally, brown microalgae, including diatoms and dinoflagellates, are highly adaptable and can thrive in varying sediment types by developing efficient mechanisms for light capture and nutrient uptake (87). This adaptability results in similar photosynthetic efficiency, electron transport rates, and light saturation rates across different sediment environments (88, 89). However, results presented here show that the relative abundances of both diatoms and dinoflagellates were higher in sand than in mud. The greater abundance of these microalgae in the sand might be attributed to better and faster drainage and oxygenation. Sand provides faster drainage and better oxygenation due to its coarser texture and greater porosity. This environment is beneficial for these microalgae, which require oxygen for their cellular processes and may be inhibited by the anaerobic conditions more common in mud (90, 91).

This study investigates the seasonal dynamics of microeukaryotic communities in intertidal sediments from Qingdao, China, by conducting monthly samplings over a year across muddy and sandy sediments between Licun and Zhanqiao Stations. Using 18S rRNA sequencing, the research analyzes alpha-diversity, OTU composition, community composition, network structure, and three trophic functional groups. The findings reveal significant monthly changes linked to environmental factors, with distinct responses in muddy and sandy environments, providing critical insights into the impacts of seasonal variations on these essential ecosystems.

### Highlights

- Significant monthly changes in community composition are closely linked to changes in environmental factors, such as temperature, dissolved oxygen, and salinity.
- Species co-occurrence network analysis shows that interaction numbers and network density peaked in summer and declined in autumn and winter.
- Responses of autotrophs, heterotrophs, and mixotrophs to seasonal environmental changes and different sediment types are described, noting peaks in abundance for autotrophs in June, heterotrophs in December, and mixotrophs from September to November.

## Data Availability

The raw sequencing data have been deposited in the NCBI Sequence Read Archive. The BioProject accession number is PRJNA1268665.

## SUPPLEMENTAL MATERIAL

Fig. S1 spectrum.00467-25-s0001.docx Alpha diversity.

ASM does not own the copyrights to Supplemental Material that may be linked to, or accessed through, an article. The authors have granted ASM a non-exclusive, world-wide license to publish the Supplemental Material files. Please contact the corresponding author directly for reuse.
