# Supplementary material for: Annual changes in the microeukaryotic community in intertidal sediments, Qingdao, China
Source: Microbiol Spectr. 2025 Oct 31;13(12):e00467-25. doi: 10.1128/spectrum.00467-25 (PMC12671089; doi:10.1128/spectrum.00467-25)
Supplement: Fig. S1 — Alpha diversity. [file spectrum.00467-25-s0001.docx]

Supplementary Files

Figure S1. Alpha diversity, species richness estimates (Chao1 and ACE). Comparison of α-diversity of microeukaryotic communities between sediment type, station and month (all *p*_months_ > 0.05, *p*_sediment_ and *p*_station_ <0.01).
